# ETHNIC VARIATIONS IN SOCIAL CAPITAL IN SOUTH ASIAN AND CHINESE OLDER ADULTS IN HONG KONG

**DOI:** 10.1093/geroni/igad104.2635

**Published:** 2023-12-21

**Authors:** Daniel W L Lai, Alison X T Ou, Vincent W P Lee, Doris S F Yu, Jia Li, Shireen Surood, Gary K K Lau

**Affiliations:** Hong Kong Baptist University, Hong Kong, Hong Kong; Hong Kong Baptist University, Hong Kong, Hong Kong; Hong Kong Baptist University, Hong Kong, Hong Kong; The University of Hong Kong, Hong Kong, Hong Kong; Chinese University of Hong Kong, Hong Kong, Hong Kong; Alberta Health Services, Edmonton, Alberta, Canada; The University of Hong Kong, Hong Kong, Hong Kong

## Abstract

The role of social capital has gained a lot of attention as one of the determinants of wellbeing of aging adults. It serves the functions of connecting people with vital resources essential to improvement of health, promotion of social cohesion and networks, and enhancement of support received. Social capital could be influenced by a variety of individual and socio-cultural factors among racialized groups. This study aims to examine the variations in social capital in Chinese and South Asian aging adults in Hong Kong. A sample of 1,015 people aged 55 and above, consisting of Chinese (n=800) and South Asian participants (n=215), participated in a survey via telephone (Chinese) and face-to-face (South Asian) interview respectively. A 25-item World Bank’s Social Capital Assessment Tool was used for measuring six variables related to social capital including social participation, social support, social connection, trust, cohesion, and reciprocity, covering the structural and cognitive dimensions of social capital. A generalized linear model was performed to detect differences between the two groups after controlling for demographics. The aging Chinese people exhibited a stronger cohesion level than their South Asian counterparts, whereas the aging South Asians demonstrated higher levels of social participation, social support, social connection, trust, and reciprocity. The identified gaps further illustrate the socio-cultural differences between the groups, highlighting the importance of strategies for enhancing social inclusion and equity for racialized older people in the Chinese context.

